# Biomechanical comparison of reverse offset‐L osteotomy and chevron osteotomy in cadaveric hallux valgus surgery

**DOI:** 10.1002/jfa2.12046

**Published:** 2024-07-28

**Authors:** Tunca Cingoz, Nezih Ziroglu, Ergun Bozdag, Fatih Yamak, Tahir Koray Yozgatli, Alp Bayramoglu, Baris Kocaoglu, Behic Tanil Esemenli

**Affiliations:** ^1^ Department of Orthopedic Surgery Acibadem University Faculty of Medicine Istanbul Turkey; ^2^ Faculty of Mechanical Engineering Istanbul Istanbul Technical University Istanbul Turkey; ^3^ Department of Anatomy Acibadem University Faculty of Medicine Istanbul Turkey; ^4^ Department of Orthopedic Surgery Istanbul Kent University Istanbul Turkey

**Keywords:** biomechanics, bunion, cadaver study, great toe, metatarsal

## Abstract

**Objective:**

Chevron osteotomy offers near‐excellent clinical results and adequate stability at lower shift percentages, among the techniques used to correct hallux valgus deformity. This cadaveric study aimed to compare the Chevron osteotomy with the reverse offset‐L osteotomy, which may provide a greater surface area and a more stable geometry to withstand higher cantilever forces at higher shift percentages.

**Methods:**

Metatarsal bones obtained from 20 human cadavers with similar bone quality were divided into two groups: Chevron osteotomy was applied to the 1st group and reverse offset‐L osteotomy was applied to the 2nd group. The load‐to‐failure, displacement in the *y*‐axis, and total displacement values of both groups were compared statistically. Furthermore, bone densities were compared between the groups with computed tomography imaging.

**Results:**

When outliers in both groups were excluded, a statistically significant difference was found in favor of reverse offset‐L (143 ± 42 vs. 204 ± 51.2 N, *p* = 0.02) in terms of failure load. The groups were similar in terms of displacement on the *y*‐axis and total displacement values. Bone densities were similar.

**Conclusion:**

The reverse offset‐L osteotomy has been shown to withstand greater loads before failure compared to the standard Chevron osteotomy. This significant difference in load‐to‐failure may enable reverse offset‐L to provide reliable stability in osteotomies performed in advanced HV cases requiring higher shifts.

## 1 INTRODUCTION

Hallux valgus (HV) is a complex deformity characterized by the lateral deviation of the big toe. Although it is more common with increasing age and in females, it is seen in almost one in every five people in the world [1, 2]. In treating moderate to severe cases of HV, generally, a first metatarsal osteotomy is used together with a distal soft tissue procedure [3]. In addition to traditional osteotomies such as Ludloff, proximal and modified Chevron osteotomies, Scarf osteotomy, and rotational variants, Lapidus first tarsometatarsal joint arthrodesis and, currently, minimally invasive distal metatarsal osteotomies are the most used techniques [3, 4, 5, 6]. Traditionally, when the HV angle is greater than 35° and the intermetatarsal (IM) angle is greater than 13°, distal osteotomies alone are considered unable to provide adequate correction with enough stability [7].

Proximal osteotomies may be preferred to overcome deformities requiring a higher shift; however, they generally necessitate longer non‐weight‐bearing periods and have some complications such as nonunion and stress fractures [8]. In comparison, distal osteotomies have the advantage of earlier weight bearing, satisfactory clinical outcomes, and lower nonunion rates and they have a wide range of indications in contemporary practice. Yet they can only be used for mild and moderate HV deformities [7, 8, 9]. Chevron osteotomy is considered one of the most stable osteotomies and has good clinical and biomechanical results [10, 11]. Despite the advantages of Chevron osteotomy, higher shifts with this osteotomy may still lead to several complications, such as instability, avascular necrosis, or malunion [12, 13]. To answer this challenge and combine the best qualities of the proximal and distal osteotomies, the reverse offset‐L osteotomy may be utilized to correct deformities requiring higher shifts by providing a larger surface area for bone healing and greater stability under vertical loads thanks to its stable geometric structure [13, 14, 15].

This study aimed to compare the standard Chevron osteotomy with the reverse offset‐L osteotomy at a high correction level regarding biomechanical stability. The study hypothesizes that reverse offset‐L osteotomy is more stable than Chevron osteotomy at high correction levels (50% shift) regarding biomechanical stability.

## 2 MATERIALS AND METHODS

Ethics approval was obtained from the local ethics committee. The first metatarsals were obtained from 21 fresh frozen human cadavers from the university's anatomy laboratory (13 from the right foot and eight from the left). All metatarsal bones used belonged to healthy feet with morphologically normal metatarsal bones and without a history of fractures or previous surgeries. The soft tissues were mechanically excised, and the bones were wrapped in a gauze dressing moistened with saline solution and kept at −20°C. The metatarsals were randomized by numbering the bones and were divided into two groups by blindly selecting numbers placed in a bag, with 10 bones assigned to each group. The remaining metatarsals at the end of the randomization were used as a control.

**FIGURE 1:**
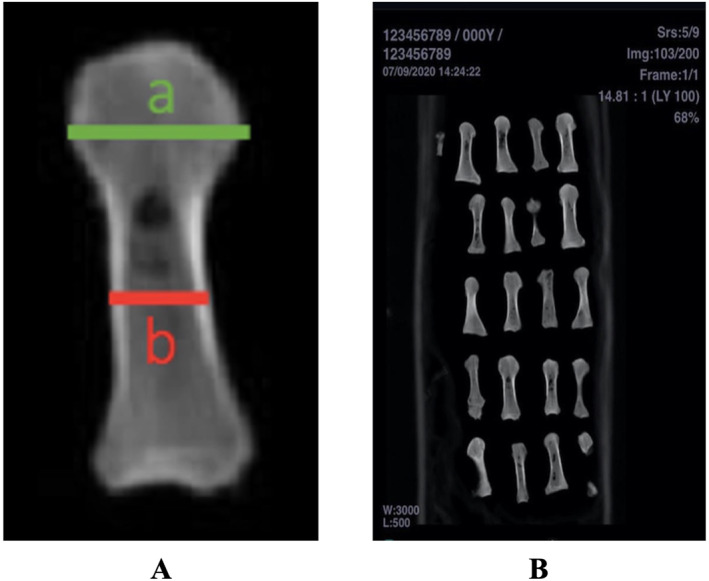
(A) The cadavers were numbered and their cortical and subcortical bone qualities were evaluated by 1 mm thin‐section computed tomography performed before the experiment. (B) All metatarsals placed on the workstation were evaluated by a radiologist specializing in the musculoskeletal system. The Region of Interest was placed by determining a 1‐cm^2^ area in the proximal, middle, and distal regions of all metatarsals, and the average densities of each of the selected areas were determined and the average Hounsfield Unit (HU) values were recorded.

There were no deformed bones as visually inspected and seen on the computed tomography (CT) scan images in the metatarsals, and cartilage tissue integrity was excellent without any visible lesions or any softening of the articular cartilage in all the first metatarsals. To assess the quality of the cortical and subcortical bone of the metatarsals, all cadavers were imaged with a 1 mm thin‐section CT imaging before the experiment. The scans of the enumerated metatarsal bones were captured using the CT (CT Optima 660 General Electric Healthcare, 2015) device with the parameters: 140 kV and 60 mA (Figure 1A). Then, all bones were evaluated one by one on the workstation (Infinitt PACS; Infinitt Healthcare) by a musculoskeletal specialist radiologist. An area of 1 cm^2^ was determined from the proximal, middle, and distal parts of each metatarsal, and a Region of Interest was placed. The mean Hounsfield Unit (HU) values were measured on the imaging workstation with the help of a computer‐aided program, and the average densities of each of the selected areas were determined and recorded (Figure 1B).

### 2.1 Surgical technique

**FIGURE 2:**
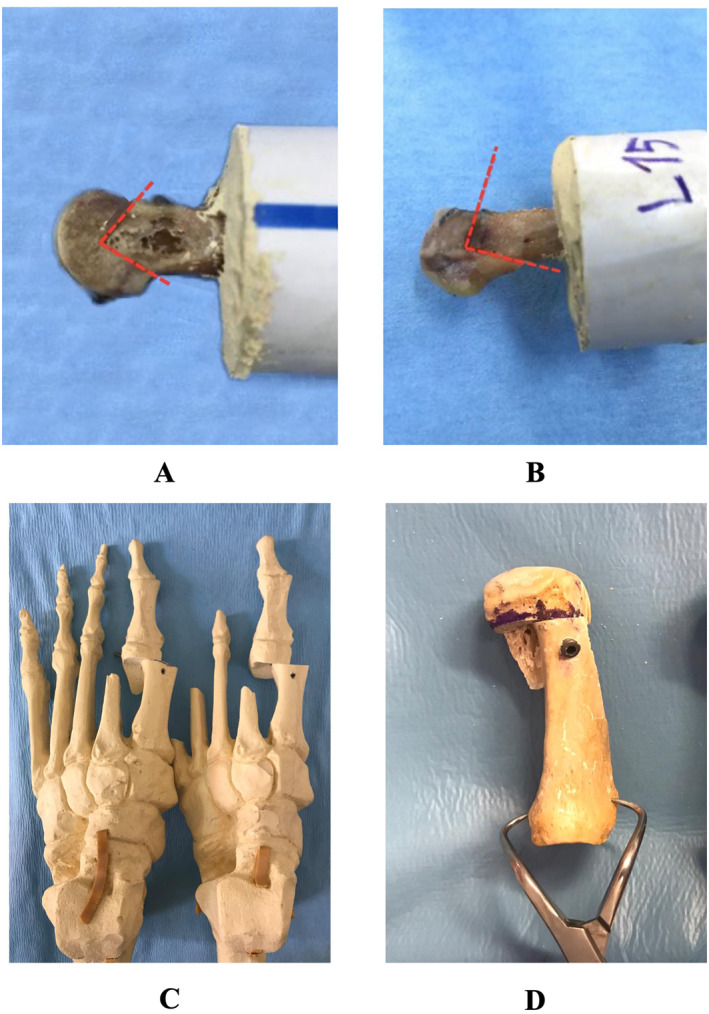
(A) Standard chevron osteotomy application performed by shifting 50% of the metatarsal width in the range of 60–80°. (B) Reverse offset‐L osteotomy application, which is performed to shift 50% of the metatarsal width within the range of 90°. (C) Axial views of metatarsals that underwent standard Chevron and reverse offset‐L osteotomies. (D) Sagittal view of metatarsals undergoing reverse offset‐L osteotomy.

In Group 1, standard Chevron osteotomy was performed as described in previous studies [16, 17, 18]. First, the largest diameter of the metatarsal head was measured in the transverse (anteroposterior) plane and marked. Then, with the help of 4 and 9‐mm saw blades, the midpoint was determined on the metatarsal head at an average of 4–6 mm proximal to the subchondral bone. From this midpoint, dorsal and plantar rays were directed at 30–40° to each side; osteotomy was performed for a total angle of 60–80°. Since the stability may decrease as the angle increases, care was taken to complete the osteotomy angle within the range of 60–80°. After the osteotomy, the distal fragment was shifted laterally to 50% of the metatarsal width (Figure 2C). Temporary fixation was then achieved with a bone clamp or manually. Then, the bone was drilled from the dorsomedial side of the proximal part to the lateral side of the distal region. Fixation was achieved with a 2.8‐mm Herbert screw (Medartis APTUS Speed Tip) after depth measurement. Afterward, the medial protrusion on the proximal portion of the osteotomy was reshaped starting from the dorsomedial toward the medial and proximal metatarsal neck with a saw appropriately in line with the distal portion (Figure 2A).

In Group 2, the reverse offset‐L osteotomy technique was performed. As in Group 1, the largest diameter of the metatarsal head was measured and marked. Then, using 4 and 9‐mm saw blades, osteotomy was started on the metatarsal head, 4–6 mm proximal and dorsal to the subchondral bone, starting from the dorsal side 10° to the vertical line, toward the midline. When the midline was reached, the osteotomy was completed by angling 90° proximally. The length of the dorsal and plantar osteotomies varied according to the metatarsal dimensions. Then, the distal fragment was shifted laterally to be 50% of the metatarsal width (Figure 2C,D). Temporary fixation was achieved with a bone clamp or manual assistance. Then, the fragments were drilled and fixed as described previously and the proximal medial protrusion was reshaped in the manner described previously (Figure 2B,D).

### 2.2 Biomechanical testing

**FIGURE 3:**
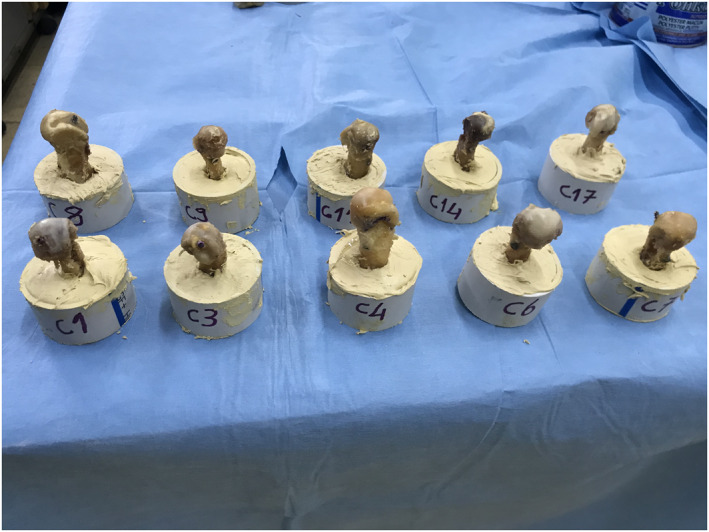
Metatarsals with their proximal ends embedded in PVC pipes with steel putty and their distal ends left exposed.

**FIGURE 4:**
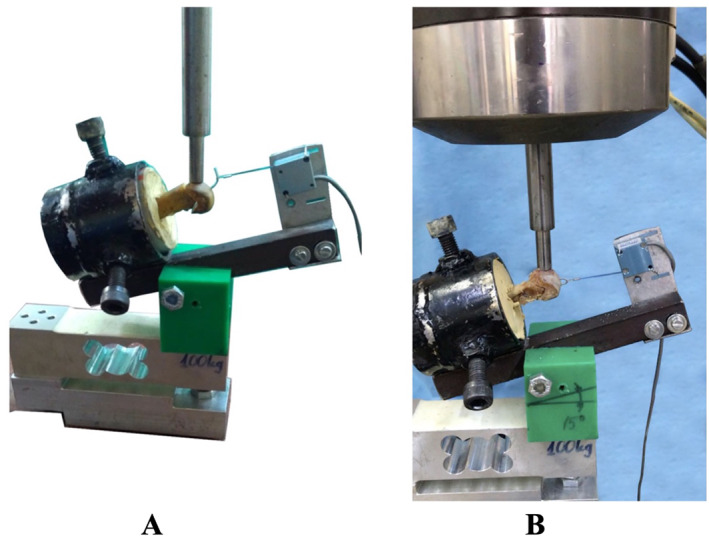
(A) Biomechanical experimental setup of reverse offset‐L osteotomy, in which a 15‐degree angle is created to simulate the natural position of the foot bone structure with the adapter attached to the load cell attached to the device. (B) Biomechanical experimental setup of standard chevron osteotomy, where a 15‐degree angle is created to simulate the natural position of the foot bone structure with the adapter attached to the load cell attached to the device.

The proximal sides of the metatarsals were embedded in the PVC conduit pipes with steel paste; the distal ends were left exposed (Figure 3). A 1.2‐mm diameter Kirschner wire was used to aid fixation, without crossing the osteotomy line. With an adapter made of cestamite material attached to a load cell (ESIT SPA 100 kg) attached to the device (MTS 858 Mini Bionix II, 2005), a 15‐degree angle was created to simulate the natural position of the bone in the foot (15, 20, 30). A steel pot was placed on this adapter, and a linear variable differential transformer (LVDT) (Unimeasure ZX Series, Portland, USA) sensor was positioned across this pot. Prepared PVC pipes were placed inside the steel pot (Figure 4).

The cable of the LVDT sensor placed across the steel pot was attached to the Kirschner wire at the bone end, thus measuring the displacement along the horizontal axis. Displacement in the vertical axis was measured with the device's own LVDT sensor. The total displacement was calculated by taking the resultant of the two displacements. The force on the bone was measured with the load cell. The dimensions of the apparatus applying force to the bone were as follows: the outer diameter was 9.0 mm, the inner diameter was 5.5 mm, and the surface area was 39.86 mm^2^. Force was applied to all specimens at a rate of 5.0 mm/min as cantilever force from the plantar face of the metatarsal head at the far end of the bone (Figure 4) [15]. The metatarsal bone without either osteotomy, kept as a control before the biomechanical test, was embedded in the PVC pipe with steel paste, and a 250‐N force was applied in the manner described above, no fracture or instability was observed. The forces were calculated using MATLAB (Mathworks) software.

### 2.3 Data and statistical analysis

Before the study, a power analysis was done according to the anticipated load‐to‐failure values, it was determined that a minimum of 8 metatarsals in each group (16 total) were needed to determine a significant difference with an alpha value of 0.05 and a beta value of 0.80. All statistical analyses were performed using R Statistical Software (R version 4.2.1; R Foundation for Statistical Computing, Vienna, Austria) and R‐Studio (Integrated Development for R. RStudio, PBC). Continuous variables were described as mean−standard deviation unless stated otherwise. Categorical variables were described as *n* (%). Continuous variables were compared with the independent samples *t*‐test for normally distributed quantitative data and the Wilcoxon rank‐sum test for nonparametric quantitative data. Categorical variables were compared by the Chi‐Square test without Yates' correction for observations with expected frequencies equal to or more than five and with Fisher's Exact Test for observations with expected frequencies less than five. Relationships between measurements were evaluated by Pearson Correlation analysis for normally distributed values and Spearman's Correlation analysis for nonparametric values. Outlier analysis was performed using the outliers R‐package as described previously [19]. For statistical significance, a *p*‐value of <0.05 was considered significant.

## 3 RESULTS

There were 10 metatarsal bones in each group. There was no statistically significant difference between the groups in terms of bone density, metatarsal head diameter, and head‐shaft diameter ratio (Table 1). The mean load‐to‐failure was greater in the Reverse Offset‐L osteotomy group; however, the difference detected was not statistically significant with the numbers available (176N ± 73.5 vs. 127N ± 50.8 N, *p* = .1002). The displacements in the *y*‐axis and total displacements were similar. Table 1 depicts the results related to direct comparisons. When the analysis was continued by removing the outliers in the data set, the load‐to‐failure analysis revealed a significantly increased load‐to‐failure in the reverse offset‐L osteotomy group. The analysis of the groups with the outliers excluded is depicted in Table 2.

**TABLE 1:** Load‐to‐failure, *Y*‐axis displacement and total displacement measurements of the chevron osteotomy and reverse offset‐L osteotomy groups.

	Chevron osteotomy (*n* = 10)	Reverse offset‐L osteotomy (*n* = 10)	*p*‐value
Load‐to‐failure (N), (mean ± SD)	127 ± 50.8	176 ± 73.5	0.10
*Y*‐axis displacement (mm), (mean ± SD)	−5.53 ± 2.14	−6.07 ± 1.36	0.51
Total displacement (mm), (mean ± SD)	6.36 ± 3.76	5.55 ± 1.59	0.79
Bone density HU (mean ± SD)	172 ± 39.7	163 ± 29.5	0.59
Metatarsal head diameter (mm), (mean ± SD)	21.32 ± 2.57	22.29 ± 2.79	0.33
Head‐to‐shaft ratio (mean ± SD)	1.81 ± 0.21	1.84 ± 0.22	0.71

Abbreviations: HU, Hounsfield Unit; mm, milimeter; N, Newton (in first column); N, number (in first row); *p*‐value <0.05: significant; SD, standard deviation.

**TABLE 2:** Load‐to‐failure, *Y*‐axis displacement and total displacement measurements of the chevron osteotomy and reverse offset‐L osteotomy groups with the outliers excluded.

	Chevron osteotomy (*n* = 8)	Reverse offset‐L osteotomy (*n* = 8)	*p*‐value
Load‐to‐failure (N), (mean ± SD)	143 ± 42.1	204 ± 51.2	0.02
*Y*‐axis displacement (mm), (mean ± SD)	−6.10 ± 2.00	−6.20 ± 1.51	0.91
Total displacement (mm), (mean ± SD)	7.02 ± 3.92	5.32 ± 1.72	0.29
Bone density HU (mean ± SD)	182 ± 37.5	173 ± 24.3	0.13
Metatarsal head diameter (mm), (mean ± SD)	21.49 ± 2.76	22.51 ± 3.11	0.56
Head‐to‐shaft ratio (mean ± SD)	1.82 ± 0.23	1.84 ± 0.23	0.85

Abbreviations: HU, Hounsfield Unit; mm, milimeter; N, Newton (in first column); N, number (in first row); *p*‐value <0.05: significant; SD, standard deviation.

When the correlation between the load‐to‐failure and bone density, metatarsal head diameter, and metatarsal head/shaft ratios were checked, there was a statistically significant positive correlation between the bone density and load‐to‐failure in both Chevron osteotomy and reverse offset‐L osteotomy groups. There was no significant correlation between the metatarsal head diameter and head/shaft ratio and load‐to‐failure values in either group with the numbers available. Table 3 depicts the results of the correlation analysis. When outliers were excluded from the analysis, there was a positive correlation between bone density and load‐to‐failure in both groups (Table 4).

**TABLE 3:** Correlation analysis of load‐to‐failure with the bone density, metatarsal head diameter, and head‐to‐shaft ratio in both groups.

Group	Parameter investigated	*R*	*p*‐value
Chevron osteotomy	Bone density	0.7335	0.016
Reverse offset‐L osteotomy	Bone density	0.9307	<0.0001
Chevron osteotomy	Metatarsal head diameter	0.0912	0.80
Reverse offset‐L osteotomy	Metatarsal head diameter	0.2863	0.42
Chevron osteotomy	Head‐to‐shaft ratio	−0.3503	0.32
Reverse offset‐L osteotomy	Head‐to‐shaft ratio	−0.3682	0.30

Abbreviations: *p*‐value <0.05, significant; r, Spearman's Correlation or Spearman's Correlation for rows with*.

**TABLE 4:** Correlation analysis of load‐to‐failure with the bone density, metatarsal head diameter, and head‐to‐shaft ratio in both groups with outliers excluded.

Group	Parameter investigated	*R*	*p*‐value
Chevron osteotomy	Bone density	0.5855	0.13
Reverse offset‐L osteotomy	Bone density	0.8936	0.003
Chevron osteotomy	Metatarsal head diameter	−0.1198	0.78
Reverse offset‐L osteotomy	Metatarsal head diameter	0.2500	0.55
Chevron osteotomy	Head‐to‐shaft ratio	−0.6181	0.10
Reverse offset‐L osteotomy	Head‐to‐shaft ratio	−0.6527	0.08

Abbreviations: *p*‐value <0.05, significant; r, Spearman's Correlation or Spearman's Correlation for rows with*.

## 4 DISCUSSION

The most important finding of the study is that reverse offset‐L osteotomy can significantly increase load‐to‐failure values compared to standard Chevron osteotomy (204 ± 51.3 vs. 143 ± 42.1 N, *p* = .0222). When outliers are excluded, this increase in load‐to‐failure with the reverse offset‐L osteotomy may be indicative of the biomechanical stability advantage of this osteotomy over the Chevron osteotomy at its high (50%) shift rate.

In cases where standard distal osteotomies may be biomechanically inadequate or instead of proximal osteotomies that can correct advanced deformities where higher shifts are required, reverse offset‐L osteotomy can be considered as an alternative [20]. In addition, despite many clinically and radiologically similar and contrast studies in the literature [21, 22, 23, 24, 25, 26], the efficacy of rotational Scarf osteotomy in severe HV cases and its possible advantages and disadvantages over conventional Scarf and distal metatarsal Chevron osteotomies, as well as Lapidus tarsometatarsal fusion, have also been demonstrated [27, 28, 29, 30].

Favre et al. presented similar results in a biomechanical comparison of Scarf, modified Chevron, and reverse offset‐L osteotomies. Consistent with our findings, the Reverse offset‐L osteotomy was able to withstand higher load values than both the modified Chevron osteotomy and the Scarf osteotomy in cantilever loading in the saw bone model [31].

In the study by Vienne et al., in which they biomechanically compared Scarf, modified Chevron, and reverse offset‐L osteotomies in sawbones, Chevron and Reverse Offset‐L osteotomies were found to be more stable [32]. Scarf and reverse offset‐L osteotomies provided more rotational resistance with the surgical method in which Chevron osteotomy was fixed with a single screw and Scarf and reverse offset‐L osteotomies were fixed with two screws. The authors also suggested that stress fractures on the proximal screw are less likely in reverse offset‐L osteotomy because it causes less stress on the proximal screw [32]. Therefore, they argued that reverse offset‐L osteotomy carries the advantages of both techniques.

In this study, no failure outside the osteotomy line or fracture around the screw was observed during biomechanical testing. Since it is known that stable osteotomies do not impose any load on the implants, the results seem to be compatible with the literature [13, 14, 15, 33]. Metatarsal head crushing was observed in two bones in each group during testing, and minimal loosening around the screw was noted in one metatarsal in the reverse offset‐L group. Nevertheless, all samples completed the tests successfully.

The contact surface area measured after Chevron and Reverse Offset‐L osteotomy in sawbones was found to be 40.5% higher in favor of Reverse offset‐L osteotomy [32]. A larger surface area can contribute positively to stability. It may also provide space where a second screw can be placed, as shared in the literature. Although it is known to increase the bone contact surface area, it was not performed in this study to minimize variables and standardize the comparison. A second screw can be applied to reduce rotational instability. A larger surface area can also prevent clinical complications such as nonunion, fixation, and implant problems.

In recent years, the number of clinical studies on the reverse offset‐L osteotomy method in HV surgery has been increasing. A study by Jentzsch et al. published the mid‐to‐long‐term results of HV patients who underwent reverse offset‐L osteotomy and reported that complication rates were low, patient satisfaction was adequate, implant removal rates were acceptable, and radiological improvement results in HV were very good [34]. Deenik et al. showed that radiological recurrence rates in reverse offset‐L osteotomy were like Chevron and Scarf osteotomies [35]. Many studies have shown that the reverse offset‐L osteotomy method is a clinically, radiologically, and biomechanically stable osteotomy method in HV surgery [15, 32, 33].

Biomechanical parameters such as cantilever loading, physiological loading, shifting amount, strain forces, cyclic loads, stress loads acting on the bone, amount of stretching, and contact surface areas are used to compare different osteotomy methods applied in HV surgery [32, 34, 35, 36, 37]. Since console loading is used for comparison purposes in many studies, this method was preferred to be used in the presented study. To determine the stability of both osteotomies, immediate and significant loss of strength reflecting the load‐to‐failure during testing was measured.

Hernandez et al. reported excellent MTP joint mobility and patient satisfaction in the long‐term results of percutaneous reverse offset‐L osteotomy in moderate HV patients [38]. The MTP joint range of motion is an area we did not study in our experiment and can be highlighted as another possible benefit of the technique.

There are several advantages of the presented study. In addition to the cantilever loading measurements, with the help of a sensor which, to the best of our knowledge, was not defined or used by any previous biomechanical studies on HV surgery, it was checked whether there was any loosening in the system during 150‐N force showing the overall stability of the system and the accuracy of the measurements were fully adequate. This measurement accuracy is one of the most important superiorities of the presented study compared to the literature. Additionally, an important superiority of the presented study is that the samples used in the biomechanical study belonged to human cadavers, whereas sawbones were commonly used in previous studies. Biomechanical studies with cadavers are superior to sawbones [39]. Performing the bone densimeter measurements on all metatarsals in both groups is another strength of the study. Densimeter measurements and comparisons were made on all cadaveric specimens to exclude invisible pathologies that could change bone quality and to distribute the groups homogeneously [11]. Groups were similar regarding bone quality.

Overall, the bone quality measurements proved important, and the bone density was positively correlated with greater load‐to‐failure in both groups (*p* < 0.05); however, when the outliers were excluded from both groups, the correlation, although still positive, was no longer significant in the Chevron group (*p* = 0.1272), which may be due to the lower number of samples with excluded outliers. The head/shaft ratio or the metatarsal head diameter and the load‐to‐failure values were not statistically significant with the numbers available.

The study has some limitations. First, isolated cantilever load measurement without subjecting the foot to rotational forces, physiological, and cyclic loads may not fully assess osteotomy stability. Additionally, since isolated metatarsal bones were used in this study, in vivo minor secondary forces of the foot could not be evaluated. Although bone densities are measured, variable cortex thicknesses, which are not evaluated, maybe another limiting factor. Due to the nature of a biomechanical study, the stability of time zero can only be understood after the surgical procedure. Finally, although the subject pool had sufficient statistical power, the analysis continued by removing outliers in the data set to ensure accurate results.

In conclusion, this study demonstrates the superiority of reverse offset‐L osteotomy over standard Chevron osteotomy in terms of load‐to‐failure with cantilever loading, at high levels of correction (50% offset). It may indicate that the reverse offset‐L osteotomy is more stable in the more advanced osteotomy shifts required to correct moderate to severe HV.

Clinical studies involving physiological soft tissue balance and performing osteotomies with higher shifts may help overcome the potential limitations of the cadaveric biomechanical study.

## AUTHOR CONTRIBUTIONS

**Tunca Cingoz**: Data Curation; formal analysis; funding acquisition; investigation; project administration; resources; software; validation; visualization; writing – original draft. **Nezih Ziroglu**: Supervision; writing – original draft; writing – review & editing. **Ergun Bozdag**: Data Curation; project administration; resources. **Fatih Yamak**: Data Curation; project administration; resources. **Tahir Koray Yozgatli**: Validation; writing – Original Draft. **Alp Bayramoglu**: Project Administration; resources; visualization. **Baris Kocaoglu**: Conceptualization; investigation; methodology; supervision. **Behic Tanil Esemenli**: Conceptualization.

## CONFLICT OF INTEREST STATEMENT

None reported.

## ETHICS STATEMENT

Ethics approval was obtained from the local ethics committee (Acibadem University Research Ethics Committee Istanbul ATADEK‐2016/20, ATADEK‐2020/23).

## Data Availability

The data that support the findings of this study are available from the corresponding author upon reasonable request.
